# Subtle Shades of Impairment: Childhood Tetrachloroethylene Exposure May Cause Subclinical Deficits in Adult Vision

**DOI:** 10.1289/ehp.120-a362a

**Published:** 2012-08-31

**Authors:** Wendee Holtcamp

**Affiliations:** Wendee Holtcamp, based in Houston, TX, has written for *Nature*, *Scientific American*, *National Wildlife*, and other magazines.

People come into regular contact with tetrachloroethylene (also known as perchloroethylene, or PCE) through dry-cleaned clothes, and the chemical has become a frequent contaminant in surface and groundwater, often due to improper disposal. PCE has been associated with neurologic and visual impairment in exposed adults. Now investigators have detected visual impairment in adults whose exposure to PCE-contaminated drinking water had occurred years earlier—from gestation through age 5 years **[*EHP* 120(9):1327–1332; Getz et al.]**.

Some 660 miles of improperly cured vinyl-lined drinking water pipes installed between 1968 and 1980 leached PCE into the water delivered to residences at different parts of the Cape Cod region of Massachusetts. Previous studies of this cohort—exposed and unexposed adults born to Cape Cod parents from 1969 through 1983—have detected evidence that PCE exposure may contribute to other neurologic impairments, including post-traumatic stress disorder, bipolar disorder, and diminished performance on tests of memory, learning, and visuospatial functioning.

In this study, the investigators tested the visual acuity (sharpness of vision), contrast sensitivity (ability to distinguish gradations of light and dark), and color discrimination (ability to correctly identify colors) in 25 exposed and 40 unexposed members of the cohort. The researchers used an algorithm to estimate PCE exposure based on each individual’s residence during gestation and early childhood, since the presence and degree of PCE contamination in drinking water varied by locale.

Tests of color discrimination revealed that high PCE exposure was significantly associated with color confusion, with all major errors occurring in blue–yellow discrimination. Only the highest PCE exposure level was associated with impaired contrast sensitivity, but these results were not statistically significant. Visual acuity did not appear to be affected; most study participants had 20:20 vision, and no differences were observed between exposure groups. The measured vision impairments are subclinical, meaning they would not have directly noticeable effects on an individual’s vision.

Previous studies have only assessed relationships between PCE exposure and vision in either childhood or adulthood. The results of this retrospective study must be confirmed, given its small sample size, but the findings suggest that childhood PCE exposure may cause long-term visual impairment. Further study of this or other exposed populations is necessary to understand the long-term effects of early-life exposure to PCE.

